# Whole-genome sequencing analysis of soybean diversity across different countries and selection signature of Korean soybean accession

**DOI:** 10.1093/g3journal/jkae118

**Published:** 2024-06-04

**Authors:** Youngbeom Cho, Jae-Yoon Kim, Seon-Kyu Kim, Seon-Young Kim, Namshin Kim, Jinhyuk Lee, Jong-Lyul Park

**Affiliations:** Department of Bioinformatics, KRIBB School of Bioscience, University of Science and Technology (UST), Daejeon 34141, Republic of Korea; Aging Convergence Research Center, Korea Research Institute of Bioscience and Biotechnology (KRIBB), Daejeon 34141, Republic of Korea; Personalized Genomic Medicine Research Center, Korea Research Institute of Bioscience and Biotechnology (KRIBB), Daejeon 34141, Republic of Korea; Department of Bioinformatics, KRIBB School of Bioscience, University of Science and Technology (UST), Daejeon 34141, Republic of Korea; Aging Convergence Research Center, Korea Research Institute of Bioscience and Biotechnology (KRIBB), Daejeon 34141, Republic of Korea; Department of Bioinformatics, KRIBB School of Bioscience, University of Science and Technology (UST), Daejeon 34141, Republic of Korea; Personalized Genomic Medicine Research Center, Korea Research Institute of Bioscience and Biotechnology (KRIBB), Daejeon 34141, Republic of Korea; Genome Editing Research Center, Korea Research Institute of Bioscience and Biotechnology (KRIBB), Daejeon 34141, Republic of Korea; Department of Bioinformatics, KRIBB School of Bioscience, University of Science and Technology (UST), Daejeon 34141, Republic of Korea; Disease Target Structure Research Center, Korea Research Institute of Bioscience and Biotechnology (KRIBB), Daejeon 34141, Republic of Korea; Department of Bioinformatics, KRIBB School of Bioscience, University of Science and Technology (UST), Daejeon 34141, Republic of Korea; Aging Convergence Research Center, Korea Research Institute of Bioscience and Biotechnology (KRIBB), Daejeon 34141, Republic of Korea

**Keywords:** genomic architectures, whole-genome sequencing, selection signature, genetic diversity, soybean

## Abstract

Soybean is an important agricultural crop known for its high protein and oil content, contributing to essential nutritional and health benefits for humans. Domesticated in China over 5,000 years ago, soybean has since adapted to diverse environments and spread worldwide. This study aimed to investigate the genomic characteristics and population structures of 2,317 publicly available soybean whole-genome sequences from diverse geographical regions, including China, Korea, Japan, Europe, North America, and South America. We used large-scale whole-genome sequencing data to perform high-resolution analyses to reveal the genetic characteristics of soybean accessions. Soybean accessions from China and Korea exhibited landrace characteristics, indicating higher genetic diversity and adaptation to local environments. On the other hand, soybean accessions from Japan, the European Union, and South America were found to have low genetic diversity due to artificial selection and breeding for agronomic traits. We also identified key variants and genes associated with the ability to adapt to different environments. In Korean soybean accessions, we observed strong selection signals for isoflavone synthesis, an adaptive trait critical for improving soybean adaptability, survival, and reproductive success by mitigating environmental stress. Identifying specific genomic regions showing unique patterns of selective sweeps for genes such as HIDH, CYP73A11, IFS1, and CYP81E11 associated with isoflavone synthesis provided valuable insights into potential adaptation mechanisms. Our research has significantly improved our understanding of soybean diversity at the genetic level. We have identified key genetic variants and genes influencing adaptability, laying the foundation for future advances in genomics-based breeding programs and crop improvement efforts.

## Teaser

This study investigated the genomic characteristics and population structures of 2,317 soybean public whole-genome sequencing data from different regions. Soybean accessions from China, Korea, and North America exhibited landrace characteristics, indicating higher genetic diversity and adaptation to local environments. In Korean soybean accession, strong selection signals for isoflavone synthesis were observed. Key genetic variants and genes influencing adaptability were identified, laying the foundation for future advances in genomics-based breeding programs and crop improvement efforts.

## Introduction

Soybean (*Glycine max*) is a major crop species that is widely cultivated throughout the world. Soybean is a rich source of proteins and lipids, a staple of the human diet for centuries (Hymowitz 2008). Soybean domestication is believed to have originated in China over 5,000 years ago, where its wild ancestor, *Glycine soja*, can still be found today (Carter *et al.* 2004; Kim *et al.* 2012). Historical records and archaeological evidence suggest that ancient Chinese farmers first domesticated and cultivated soybeans between 3,000 and 1,000 BC (Guriqbal Singh 2010). Soybeans gradually spread from China to neighboring countries, such as Korea and Japan, where they were introduced around the first century AD (Lee *et al.* 2007). During this period, soybeans were localized into various landraces (Carter *et al.* 2004; Bandillo *et al.* 2017). Soybeans later spread to North America in the 18th century AD (Wilson 2008). In the past 50 years, global soybean production has experienced an exponential surge, exceeding 13 times the levels recorded in the early 1960s (Ritchie and Roser 2021). As soybean production continues to expand, there has been a corresponding increase in the emphasis on research related to the genetic resources of both food products for the human diet and industrial applications, such as bio-diesel (Demorest *et al*. 2016), demonstrating the broad utility of soybeans.

Soybean has adapted to a wide range of environments, including varying temperatures, moisture levels, and geographical regions from fertile plains to mountainous areas (Li *et al.* 2008). The landrace soybean accessions exhibit distinctive adaptive characteristics and possess considerable local diversity (Harlan 1975). Landrace soybean has effectively adapted to these different environments by accumulating numerous genetic variants in all traits (Dongjin *et al.* 2008). In addition, landrace soybean has developed the ability to enhance yield stability, improve fitness, and withstand both biotic and abiotic stressors (Sedivy *et al.* 2017). The emergence of landrace soybean accessions with diverse genetic characteristics is a clear indication of soybean domestication (Hyten *et al.* 2006). These adaptive traits have been achieved through accumulating multiple genetic variants and developing specific genetic mechanisms to cope with environmental stresses. These genetic changes have allowed soybeans to adapt to different environmental conditions and have contributed to their success as domesticated crops. Genetic diversity within landrace soybean accessions is also an essential resource for the continued improvement of soybean crops, providing a genetic resource that can be used to develop new cultivars with improved traits.

Previous studies have been done extensively on soybeans focusing on identifying differences in genetic variation and adaptive traits. These studies have primarily utilized soybean accessions from China and the United States. A previous study investigated the genetic relationships between Chinese and US soybean germplasm using single-nucleotide polymorphisms (SNP) markers (Liu *et al.* 2017). Another study investigated the genetic and genomic characteristics that allow soybeans to adapt to tropical regions by analyzing soybeans from China, South Asia, and the Americas (Dong *et al.* 2021). Furthermore, an analysis of the genome sequences of 2,214 Chinese soybeans to explore the genomic signatures of soybean evolution (Li *et al.* 2022).

Recent research has highlighted the nutritional benefits of soybeans, particularly their isoflavone content. Previous study suggested that a soy-based diet may protect against certain cancers and cardiovascular diseases (Fan *et al.* 2022). The United States Department of Agriculture (USDA) food database for isoflavone content has shown that the Korean soybean accessions have the highest isoflavone content among those from China, Japan, Europe, Brazil, and the United States (Bhagwat *et al.* 2008).

Korean soybean research is essential for addressing local needs preserving cultural traditions and ensuring food security within South Korea (Kim 2019), while Chinese and American soybean research have enormous global impacts due to their production volumes and export markets. Korean soybean research focused on genetic diversity (Jeong *et al.* 2019), domestication (Kim *et al.* 2021), nutritional quality (Park *et al.* 2022) and meeting the demands of the local market, making it significant within its context. South Korea is actively conserving its wild soybean genetic resources and utilizing them for genome-assisted functional gene discovery and soybean improvement under a National Rural Development Administration (RDA). With its large amount of wild soybean resources (Nawaz *et al.* 2020), South Korea is well positioned to contribute to global efforts to address the challenges faced by agriculture due to climate change. Given the increasing importance of Korean soybeans, it is essential to conduct research on their genomic architecture and selection analysis.

In our study, we conducted a comprehensive analysis of public whole-genome sequencing (WGS) data derived from 2,317 soybean accessions, sourced from a diverse range of countries and continents, such as China, Korea, Japan, Europe, North America, and South America. These data included 1,933 *Glycine max* and 384 *Glycine soja*. Whole-genome sequencing is a comprehensive approach that provides a detailed and complete view of an organism’s genetic makeup by analyzing entire genomes. Whole-genome sequencing allows the identification of key genetic variations and selection signatures, providing a more comprehensive view of genetic variation within and between populations compared to traditional sequencing methods that only target specific genome regions. We aimed to identify the genomic architectures and population structure of Korean soybean accessions, understand their local adaptation and selection signature better and determine key variants/genes using comparative genome-wide analysis.

## Materials and methods

### Soybean sample collection and preparation

**Fig. 1.**
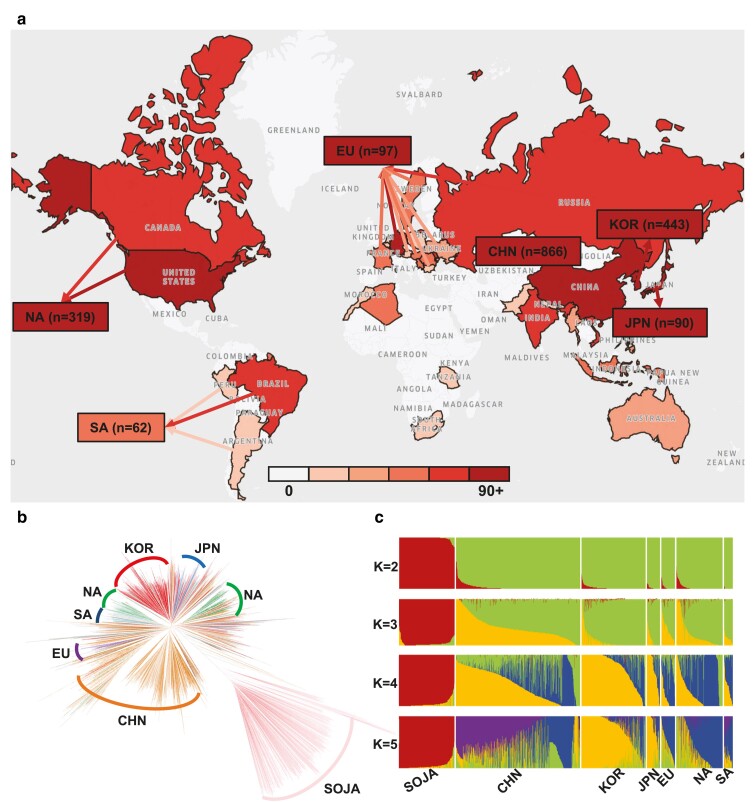
The geographical distribution of soybean samples a) A global representation highlighting the count of soybean samples procured from each country with the intensity of the color signifying the quantity of the samples. b) Genetic relationship tree among seven soybean groups and represented distinct colors indicating their respective geographic regions. c) A depiction of the population structure ranging from K=2 to K=5 categorized by geographic region.

This study aimed to investigate a comprehensive analysis of soybean population structure and detect signs of selection on a global scale by collecting large-scale whole-genome sequencing data of soybean accessions. A total of 2,317 soybean accessions were included in the study which was collected from diverse geographic regions worldwide including China, Korea, Japan, Europe, North America, South America, Oceania, and Africa. To obtain this dataset, the study utilized publicly available data from the Sequence Read Archive (SRA) (Leinonen *et al.* 2010) which included 1,933 samples of *Glycine max* and 384 samples of *Glycine soja*. The samples were grouped based on their geographic origin resulting in a distribution of 866 samples from China, 443 from Korea, 90 from Japan, 97 from Europe, 319 from North America, 62 from South America, and 56 from various other countries and continents (Fig. 1a).

### Genomic data processing and variant calling

We conducted a per-base sequence quality check on raw FastQ files from 2,317 soybean accessions using FastQC (Andrews *et al.* 2010). The sequencing reads were aligned to the Glycine max Wm82.a4v1 reference genome (Valliyodan *et al.* 2019), using the Burrows–Wheeler Aligner- (BWA) v0.7.12 (Li and Durbin 2010). The Variant Calling Pipeline, which includes the Preprocessing, filtration, and variant calling steps, has been made publicly available on GitHub (https://github.com/skyclub3/SoyPopGeneticsPipeline).

The mapping step excluded the chloroplast and mitochondrial genomes. The mapped files were sorted into the genomic coordinates of the Wm82.a4.v1 genome using the “AddOrReplaceReadGroup” option within Picard software v2.0.1, and potential PCR duplicates were eliminated using the same software’s “MarkDuplicates” option (Picard 2019).

The Genome Analysis Toolkit v3.7 (GATK) was used to correct misalignments resulting from insertion/deletion polymorphisms (INDELs) by applying the “RealignerTargetCreator” and “IndelRealigner” options. Next, gVCF files for 2,317 samples were generated using the “HaplotypeCaller” option. These samples were called from all base sites of the reference genome and combined into a single gVCF file using the “CombineGVCFs” option. This file was then converted into a single VCF file using the “GenotypeVCFs” option. We employed specific options for the GATK “VariantFiltration” and “SelectVariants” arguments to reduce the likelihood of false-positive variants. These options included: (i) quality score by depth <3.0; (ii) Phred-scale quality score <30.0; (iii) mapping quality score <30.0; (iv) genotype quality score <10.0; (v) depth of coverage across all samples <7.0; (vi) Phred-scale *P*-value score of the Fisher exact test for strand bias >30.0; (vii) rank sum test for bias of relative positions of the reference and alternative alleles <2.0; and (viii) rank sum test for mapping quality of reference and alternative reads <2.0. The VCF file quality score distribution used to determine all filtration options is summarized in Supplementary Figure 1.

In addition, to ensure the reliability of our data and to exclude outlier variants, we removed any variants exhibiting a missing genotype rate exceeding 15% in Supplementary Figure 2. The variants obtained from a stringent quality filtering process were separated into indel and SNP variants. Multi-allelic SNPs were represented by only the allele with the highest frequency, thereby creating as many bi-allelic SNPs as possible to maximize the coverage of all 2,317 soybean accessions with bi-allelic SNPs. The bi-allelic SNPs were subjected to haplotype phasing and imputation using BEAGLE v5.2 (Browning and Browning 2007), and subsequently, only bi-allelic SNPs having a minor allele frequency (MAF) greater than 1% were retained (Supplementary Figure 2). Afterward, the functional impact of these bi-allelic SNPs on genomic regions was determined by annotating them through SnpEff v4.3 (Cingolani *et al.* 2012), which utilized the Wm82.a4.v1 gene set.

### Genomic relationship and population structure

To understand the genetic relationships among soybean regional subpopulations, we reconstructed a genetic relationship tree using the Maximum-Likelihood method implemented in MEGA-X software (Kumar *et al.* 2018). This analysis was conducted on a filtered VCF file before applying an MAF filter. Genetic relationship tree analysis allowed us to estimate the evolutionary relationship between the subpopulations (Fig. 1b).

**Fig. 2.**
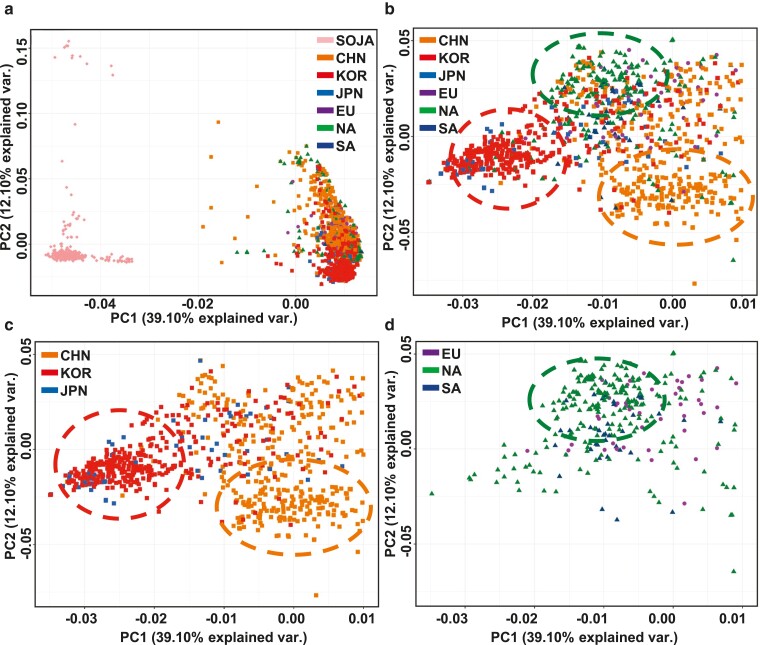
PCA Plots of Soybean Genetic Diversity a) PCA plots representing all 2,317 soybean accessions computed based on genetic diversity. b) PCA plot exclusively for *Glycine max* soybean. c) PCA plots specifically for Asian soybean groups. d) PCA plots for European and American soybean groups.

Next, we employed fastSTRUCTURE v1.0 (Raj *et al.* 2014) to perform an admixture analysis with filtered VCF file prior to MAF filter. fastSTRUCTURE employs a variational Bayesian framework to infer the number of genetic clusters (K) from 2 to 5, which is calculated using the corresponding cross-validation errors in Fig. 1c. For principal component analysis (PCA), we utilized Plink (Purcell *et al*. 2007) to obtain eigenvalues and eigenvectors from the filtered VCF file prior to MAF filter. The resulting output was visualized using R software (Fig. 2). To investigate population relationships while incorporating the effects of gene flow and genetic drift, we utilized TreeMix v1.12 (Pickrell and Pritchard 2012) to construct a maximum-likelihood tree from the filtered VCF file prior to applying MAF filter. SOJA was designated as the root population for the gene flow analysis. We employed a block size of 500 kb to infer the covariance matrix, accounting for the Linkage Disequilibrium (LD) analysis.

We employed ANGSD (Korneliussen *et al.* 2014) to analyze nucleotide diversity within each soybean accession and genetic differentiation among soybean regional subpopulation. Watterson’s theta ($\theta_{W}$) (Watterson 1975) was calculated to estimate nucleotide diversity at genome-wide averages. Pairwise fixation index value (Fst) (Weir and Cockerham 1984) was computed using angsd-wrapper (Durvasula *et al*. 2016) for a comprehensive analysis of genetic variation. To ensure accurate estimates of genetic diversity, we employed realigned BAM files generated by the GATK pipeline’s RealignerTargetCreator and IndelRealigner prior to applying VCF filteration and MAF filter. We then utilized ANGSD’s ability to analyze per-base pair data for calculating both Watterson’s theta and Fst.

**Fig. 3.**
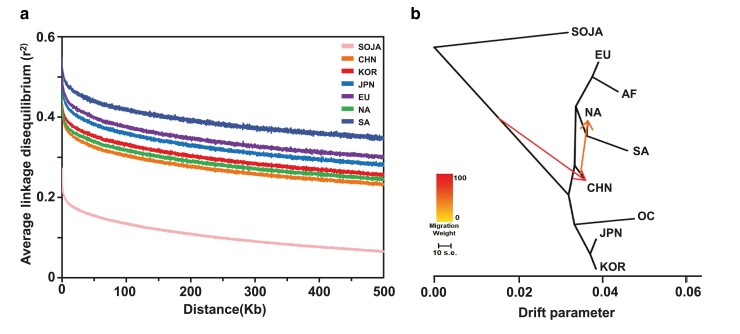
Gene Flow and LD in Soybean a) Calculation of LD decay up to a distance of 500 kb. b) The *x*-axis denotes the intensity of genetic drift. Arrows represent gene flows and migration rates derived from nine geographical soybean subpopulations.

To investigate the genetic patterns of soybean regional subpopulations, we analyzed both inbreeding coefficients (Weir and Cockerham 1984) and LD. We estimated the inbreeding coefficient (Weir and Cockerham 1984) for each soybean accession by averaging the values obtained from all samples using VCFtools v4.2 (Danecek *et al.* 2011). The inbreeding coefficient reflects the probability that an individual inherits identical alleles at a specific locus from a recent common ancestor. We then proceeded to analyze LD within the soybean accessions. LD describes the non-random association of alleles at different loci. The average LD values for each region were summarized, and the overall decay of LD with increasing distance is depicted in Fig. 3a. Notably, inbreeding coefficient and LD analysis was performed using filtered VCF file prior to applying MAF filter.

### Selection signal identification and pathway analysis for positive selection

To identify genetic loci under positive selection, we used the cross-population composite likelihood ratio test (XP-CLR) (Chen *et al.* 2010) and the cross-population extended haplotype homozygosity test (XP-EHH) (Sabeti *et al.* 2007) to detect selection signals. These tests were applied to a VCF file that had undergone BEAGLE imputation and MAF filter to enhance the detection of stronger selection signals. We utilized XP-CLR v1.0 with a size of 50-kb and a 10-kb sliding window to analyze the Whole-genome sequencing dataset. Within each window, a maximum of 2,500 SNPs were compared based on their composite likelihood scores, SNP pairs exhibiting LD greater than 0.95 were assigned lower weights, to minimize dependence effects on the scores. Due to the absence of a complete genetic map, physical positions (1Mb=1cm) were used to represent genetic positions. Selection regions were identified by selecting the top 1% of the empirical distribution of raw XP-CLR scores. These regions were then assigned to candidate gene regions.

To identify differential selection signatures between two populations, we utilized the rehh v3.2.1 (Gautier and Vitalis 2012) from R software package. Specifically, we calculated a raw score of XP-EHH, a method that compares the extended haplotype homozygosity of selected genetic loci in each population. The top and bottom 1% of the empirical distribution of XP-EHH genetic regions were accepted as selection candidate regions based on the *P*-value. Candidate genes from XP-CLR and XP-EHH were determined based on their closest to the identified candidate gene regions. Genes that covered more than 80% of either side of these candidate gene regions were also considered as candidate genes. Functional information of candidate genes was identified through gene set enrichment analysis, in which candidate genes were pooled together. The Kyoto Encyclopedia of Genes and Genomes (KEGG) pathway gene enrichment analysis was used to link candidate genes to gene functions, diseases, and biological pathways, and they were grouped based on pathway categories. The statistical significance level for the pathway categories was determined using the Bonferroni method with a *P*-value of 0.05.

## Results

### Sample preparation

In our study, we collected whole-genome sequencing on a total of 2,317 soybean samples from various regions including China (CHN), Korea (KOR), Japan (JPN), Europe (EU), North America (NA), South America (SA), Oceania (OC), and Africa (AF) (Fig. 1a). We used publicly available data from SRA including 1,933 *Glycine max* and 384 *Glycine soja* (SOJA) samples. The soybean samples were categorized based on their origin resulting in a distribution of 866 samples from CHN, 443 from KOR, 90 from JPN, 97 from EU, 319 from NA, 62 from SA, and 56 from various other countries and continents (Supplementary Table 1 and File 1).

A total of 276.7 billion reads of varying lengths (151 bp to 302 bp) were generated resulting in 41.8 Tb of sequence data aligned to the soybean reference genome, the *Glycine max* genome (Gmax_508_Wm82_a4.v1) (Valliyodan *et al.* 2019). The genome coverage of the samples averaged at 97.9% while the average read depth reached 18.6X after eliminating duplicate reads.

The variant calling pipeline identified 4,691,119,648 raw variants (Supplementary Table 2). Of these, 2,159,829,878 were INDELs, and 2,254,198,772 were SNPs. After the filtering, a total of 46,620,786 bi-allelic SNPs were retained for further population genetic analysis. These SNPs were selected based on an MAF greater than 1% and a missing rate lower than 15%.

The number of bi-allelic SNPs used in this study was relatively high compared to the previous studies (Wu *et al.* 2019; Kim *et al.* 2021) due to the larger number of samples (SOJA: 9,223,755; CHN: 7,798,075; KOR: 7,342,058; NA: 6,649,742) with SOJA having the highest number of bi-allelic SNPs (Supplementary Table 3 and Figure 3). The SOJA showed a nearly threefold increase in the number of bi-allelic SNP compared to JPN, EU, and SA accessions, which can be attributed to the concerted effort to maintain homogeneity during domestication in JPN, EU, and SA accessions (JPN, 3,512,283; EU, 2,930,790; SA, 3,059,564).

As shown in Supplementary Figure 3, the domestication process has led to a reduction in the number of genetic variants resulting in CHN, KOR, and NA regions having a higher number of bi-allelic SNPs than other regions indicating a higher degree of conserved genetic diversity in these regions. To evaluate the genomic diversity of soybean groups, we analyzed 46,620,786 bi-allelic SNPs with an MAF greater than 1% at the high genome-wide resolution covering all 2,317 samples.

### Genomic architecture of soybean

Soybean domestication, initially in Asia, has resulted in a series of genetic bottlenecks. This process began with the cultivation of a wide variety of Asian landraces and was followed by introducing these landraces to North America (Hyten *et al.* 2006). The bottleneck effect observed during the domestication process led to a swift and substantial decrease in the count of rare alleles, diminishing the genetic diversity of crops subsequently (Tanksley and McCouch 1997).

Watterson’s theta ($\theta_{W}$) is a statistical metric in population genetics, representing genetic diversity, calculated using polymorphic sites in the DNA sequence, providing a genome-wide average of genetic variation. $\theta_{W}$ analysis of 2,317 core accessions showed that SOJA exhibited the highest $\theta_{W}$ at $1.94\times 10^{4}$ with CHN and KOR displaying moderate levels of $\theta_{W}$ at $1.75\times 10^{4}$, $1.50\times 10^{4}$. JPN, EU, NA, and SA fell into the lower group exhibiting $\theta_{W}$ of $1.20\times 10^{4}$, $1.18\times 10^{4}$, $1.01\times 10^{4}$ and $0.92\times 10^{4}$ respectively (Supplementary Table 4). The $\theta_{W}$ values for CHN and KOR accessions, which are $1.75\times 10^{4}$, $1.50\times 10^{4}$ respectively indicate a higher level of genetic diversity in these soybean accessions compared to others such as JPN, EU, NA, and SA. In population genetics, a higher $\theta_{W}$ value indicates more segregating sites, locations in the DNA sequence where variations occur. This suggests that the CHN and KOR accessions have a larger number of these variable sites indicating higher genetic diversity.

The level of LD serves as an additional indicator of genomic architecture, encompassing different evolutionary influences such as genetic bottlenecks and selection pressures. The average value of LD within a 500 kb region was the highest for SA (0.49) followed by EU (0.42), JPN (0.42), KOR (0.40), NA (0.39), CHN (0.38), and SOJA (0.21) (Fig. 3a and Supplementary Table 4).

In our study, we observed that the inbreeding coefficient (Weir and Cockerham 1984) for all soybean accessions ranged from 0.86 to 0.90, while SOJA (0.86) had the lowest inbreeding coefficient, EU (0.90) and JPN (0.90) had the highest. The remaining soybean accessions, namely CHN (0.88), KOR (0.88), NA (0.88), and SA (0.89), exhibited inbreeding coefficients within this range (Supplementary Table 4).

An examination of the genomic architecture of soybeans revealed subtle variations across different geographical regions. Specifically, the CHN and KOR accessions exhibited a relatively high value of $\theta_{W}$ and a low level of LD. In contrast, the soybeans from EU, JPN and SA accessions displayed a lower genetic diversity, coupled with a comparatively higher LD and inbreeding coefficient. Furthermore, the KOR accessions not only exhibit the distinctive characteristics of landrace species, but also display the potential for unique genetic characteristics potentially valuable for breeding programs.

### Population structure and relationship

Various methods were employed to assess genetic differentiation and population structure, including PCA, admixture analysis, genetic relationship tree, and calculation of the Fst. We analyzed 2,317 soybean accessions from 10 regional subpopulations, mainly focusing on seven soybean regional groups due to the sample size. We conducted a comprehensive population analysis to investigate the population structures and relationships.

The genetic relationship tree analysis revealed that the SOJA formed a separate cluster from *Glycine max* subdivided to each country (Fig. 1b). The first cluster represented SOJA, the ancestor of the *Glycine max*. The KOR and JPN accessions are largely overlap with a minority of CHN accessions. The NA accessions are divided into two clusters, one of which is closely clustered with SA accessions. Meanwhile, the CHN accessions are more widely distributed and form the largest clade (Fig. 1b). Also, the KOR accessions formed a particularly tight cluster, suggesting they possess unique genetic traits that likely evolved through minimal genetic exchange with other soybean subpopulations (Fig. 1b).

The population structure analysis using admixture analysis (Fig. 1c) revealed that each soybean accession had a unique genetic composition. At K=2, *Glycine soja* and *Glycine max* were clearly separated. When examined from K=3 to K=5, the admixture structure differed among accessions from different countries. Based on the results of the admixture analysis, the SOJA primarily exhibited genetic characteristics that were representative of the ancestral genetic structure. The genomic composition of the KOR accessions started to show a unique composition distinct from other regions after K=2.

PCA of Glycinemax accessions revealed fascinating patterns of genetic variation. The PCA distinctly separated Glycinemax from its wild ancestor, Glycinesoja highlighting the genetic divergence that has transpired through domestication (Fig. 2a). Interestingly, within the domesticated Glycinemax, the PCA significantly overlapped various regional subpopulations (Fig. 2b). This overlap suggests shared genetic similarity among these subpopulations, potentially attributable to factors such as gene flow and shared ancestry. Our study observed a propensity for accessions from specific regions such as CHN, KOR, and NA to form clusters when sufficient samples were available (Fig. 2c–d). The distinct clustering of CHN, KOR, and NA observed in the PCA analysis provides compelling evidence of their unique genetic characteristics.

We further researched on the gene flow and genetic drift among the soybean accessions as shown in Fig. 3b. Our gene flow analysis clarified the genetic split and drifts among soybean accessions. We observed a genetic split between NA and SA that originated from CHN, along with a divergence between EU and AF. On the opposite branch, we detected a distinct genetic drift that segregates from CHN to KOR and JPN which exhibit a close divergence. A unique genetic drift was also observed influencing OC.

Pairwise Fst analysis of soybean accessions revealed distinct genetic differentiation between KOR accessions and their geographically close counterparts from CHN and JPN. This was indicated by higher Fst values between KOR and CHN ($2.42\times 10^{-2}$) and KOR and JPN ($2.55\times 10^{-2}$) compared to the lower Fst value observed between CHN and JPN ($2.14\times 10^{-2}$) (Supplementary Table 5). These findings suggest significant genetic differentiation of KOR accessions from their geographically proximate counterparts, CHN and JPN.

In conclusion, this study employed various methods to analyze the genetic diversity and population structure of the soybean regional subpopulation. Distinct genetic patterns were observed among regional subpopulations, suggesting shared ancestry, and potential gene flow. KOR accessions exhibited unique genetic traits despite their geographical proximity to CHN and JPN, likely due to their adaptation to the Korean peninsula over time.

### Dection of selection signal in KOR soybean accessions

**Fig. 4.**
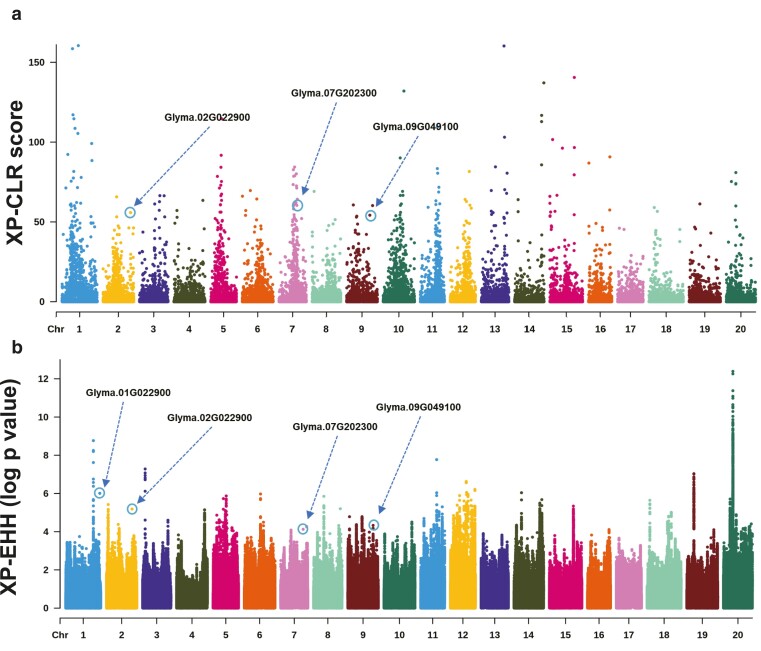
The Manhattan plot offers a comprehensive visualization of the a) XP-CLR and b) XP-EHH results obtained from the Korean soybean accession. The locations of Glyma.01G22900, Glyma.02G022900, Glyma.07G202300, and Glyma.09G49100, which are associated with isoflavone synthesis in Korean soybeans as revealed through KEGG pathway analysis, are distinctly marked on the plot.

Crop improvement naturally involves the selection of favorable alleles at genes associated with important agronomic traits, which can result in a detectable signature of selection within the genome of cultivated soybean. Identifying these selection signatures can be important for evolutionary biology and understanding the genetic architecture of agronomic traits (Wen *et al.* 2015). Farmers and breeders have developed many crop populations with adaptive traits, making them valuable reservoirs of genetic diversity. It can be leveraged to enhance the performance of other populations in diverse breeding programs (Hyten *et al.* 2006; Dwivedi *et al.* 2016). Keeping this in mind, we employed XP-CLR (Chen *et al.* 2010) method to identify genomic regions exhibiting significant allele frequency differentiation as well as XP-EHH (Sabeti *et al.* 2007) method to detect differences in haplotype structure across the SOJA, CHN, KOR, JPN, EU, NA, and SA accessions genomes to identify selection signatures of KOR accessions. XP-CLR and XP-EHH methods exhibit greater resilience towards ascertainment bias in SNP discovery compared to allele frequency spectrum-based methods. Moreover, these methods offer enhanced statistical power when compared to other approaches such as CLR, Fst, and Tajima D (Kim and Stephan 2002; Chen *et al.* 2010). Furthermore, this method has been widely employed in previous studies to identify regions of a selective sweep in crop species including soybean (Zhou *et al.* 2015; Duan *et al.* 2022), wheat (Wang *et al.* 2022), and rice (Xie *et al.* 2015; Qin *et al.* 2021). After conducting the selection analysis, a stringent cutoff was established to minimize the inclusion of false-positive regions. XP-CLR and XP-EHH are geared towards detecting deviations from neutrality. Such deviations could potentially be attributed to selection processes. Regions with extreme values comprising the top 1% of the empirical distribution for both XP-CLR and XP-EHH statistics were identified as candidate regions under selection (Fig. 4). Genes situated within specific regions were classified as potential candidate genes. In our study, we identified 562 and 2,077 such genes respectively. To discern the biological pathways linked with this set of candidate genes, we conducted an enrichment analysis using the KEGG pathway. The isoflavone synthesis pathway emerged as a common factor in both XP-EHH and XP-CLR. A total of 15 unique candidate genes were detected in XP-CLR and XP-EHH (Table 1). Additional details regarding the KEGG pathway gene enrichment analysis can be found in Supplementary File 2.

**Table 1.** Selected candidate genes from the XP-EHH and XP-CLR statistics for the Korean soybean highlighting overlapping KEGG pathway such as isoflavone synthesis (P-value<0.05).

KEGG pathway	Pathway reference	No. of related genes	*P*-value	Selected genes
XP-EHH				
Isoflavonoid biosynthesis	https://www.genome.jp/entry/gmx00943	13	1.03E-02	GLYMA_07G202300/GLYMA_03G143700/ GLYMA_08G089400/GLYMA_08G089500/ GLYMA_10G250300/GLYMA_02G236500/ GLYMA_09G049100/GLYMA_13G173300/ GLYMA_13G173600/GLYMA_09G049200/ GLYMA_16G149300/GLYMA_09G049300/ GLYMA_01G239600
Flavone and flavonol biosynthesis	https://www.genome.jp/entry/gmx00941	5	1.71E-02	GLYMA_05G021800/GLYMA_05G021900/ GLYMA_05G022100/GLYMA_13G289000/ GLYMA_13G289100
XP-CLR				
Isoflavonoid biosynthesis	https://www.genome.jp/entry/gmx00943	3	4.07E-02	GLYMA_02G236500/GLYMA_07G202300/ GLYMA_09G049100

At the genomic level, selection processes can alter the neutral patterns predicted by the neutral theory of molecular evolution, including changes in $\theta_{W}$, LD, and allele frequency (Ross-Ibarra *et al.* 2007). Under the influence of directional selection, nucleotide, and haplotype diversity decrease while LD increases (Qanbari *et al.* 2010). Also, the hitchhiking theory suggests that when selection pressure affects an allele at a specific locus, it can also impact alleles at closely linked loci (Smith and Haigh 1974; Nielsen *et al.* 2005; Gianola *et al.* 2010). This phenomenon, known as selective sweep, can form long-range haplotypes with low diversity (Sabeti *et al.* 2007). To further clarify the selection signatures, we investigated the genomic characteristic of candidate genes that exhibited exceptional values in the XP-CLR and XP-EHH statistics specifically those within the highest 1% of the empirical distributions. We measured long-range haplotype by evaluating the degree of haplotype sharing. This was achieved by visualizing the major and minor alleles of the gene region as red and yellow respectively. We investigated the hitchhiking process in regions containing missense variants and their surrounding 14 variants by examining the haplotype structure and haplotype frequencies within these regions. Our analysis revealed unique selection patterns in the HIDH, CYP73A11, IFS1, and CYP81E11 candidate genes based on the selection signals of the KOR accessions (Table 2 and Supplementary Figures 4–5). These genes are associated with isoflavonoid synthesis as shown by the KEGG pathway gene enrichment analysis.

**Table 2.** Summary of major candidate genes classified from XP-EHH and XP-CLR in Korean soybean accession.

Selected gene	ORF names^a^	Protein names	Association	XP-CLR	XP-EHH^b^	Reference
HIDH	GLYMA_01G239600	2-hydroxyisoflavanone dehydratase	Isoflavonoid biosynthesis	–	6.00	Livingstone *et al.* (2011)
C4H	GLYMA_02G236500	Cinnamate-4-hydroxylase	Flavonoid biosynthesis	52.93	5.19	Dastmalchi *et al.* (2016)
IFS1	GLYMA_07G202300	Isoflavone synthase 1	Isoflavonoid biosynthesis	60.23	4.12	Subramanian *et al.* (2004)
CYP81E11	GLYMA_09G049100	Cytochrome P450 monooxygenase	Isoflavonoid biosynthesis	46.25	5.20	Liu *et al.* (2003)

^a^ORF stands for Open Reading Frame.

^b^The maximum values of log *P*-value for the XP-EHH statistics derived from the empirical distribution.

### Adaptation of Korean soybean to isoflavonoid synthesis

Isoflavones play a vital role in plant adaptation to their biological environment. They have protective effects against various environmental stresses, such as drought and extreme temperatures (Trush and Pal’ove-Balang 2023). Isoflavones act as phytoalexins (Treutter 2005; Sohn *et al.* 2021). Phytoalexins are defensive compounds produced by plants when they are attacked by pathogens or other stressor factors. They are part of the immune system and inhibit or kill the growth of pathogens (Hammerschmidt and Nicholson 1999). Our research identified a unique signature of selection in the HIDH, CYP73A11, IFS1, and CYP81E11 genes based on the selection signals of the KOR accessions (Supplementary Figures 4–5). These genes play a role in isoflavonoid synthesis as demonstrated by the KEGG pathway gene enrichment analysis (Supplementary File 2). In our study, we discovered that the KOR accessions exhibit a strong selection signal for isoflavone synthesis. Our findings provide further support for the previous research indicating that the KOR accessions possesses the highest isoflavone content among soybeans from China, Japan, Europe, Brazil and the United States (Bhagwat *et al.* 2008).

HIDH is an enzyme that plays a key role in producing isoflavones in plants, including soybean. It catalyzes the conversion of 2-hydroxyisoflavanone to 2,3-dehydro-2,3-dihydroxyisoflavanone which is an intermediate step in the biosynthesis of isoflavones. This intermediate can then be further converted into various isoflavone molecules by other enzymes, such as isoflavone synthase (Akashi *et al.* 2005). Between 56.3464 Mb and 56.3483 Mb positions in HIDH, the KOR accessions displayed the lowest nucleotide and haplotype diversity patterns. It also exhibited a relatively long stretch of homogeneous haplotype patterns that were distinct from other soybean accessions (Supplementary Figure 4). CYP73A11 is an important enzyme in the biosynthesis of isoflavones in soybean, also known as cinnamate 4-hydroxylase (C4H) (Zhang, Zhang, *et al.* 2022). C4H plays an important role in the biosynthesis of isoflavones by converting cinnamic acid to p-coumaric acid which is a precursor to 4-coumaroyl CoA. This conversion is an important step in the production of various isoflavones such as genistein, daidzein, and glycitepin (Zhang, Zhang, *et al.* 2022). It catalyzes a key step in the biosynthetic pathway leading to the formation of naringenin chalcone which is then converted to various isoflavone molecules. The activity of CYP73A11 is regulated by various factors and plays a critical role in the plant’s response to the environmental stresses. In CYP73A11, the KOR accessions exhibited the least diverse nucleotide and haplotype patterns and also displayed a unique long-range haplotype pattern (Supplementary Figure 4). IFS1 is one of two isoflavone synthase genes in soybean. It encodes a protein that facilitates the transformation of liquiritigenin and naringenin into the specific isoflavone aglycones. They correspond to namely daidzein, glycitepin, and genistein (Anguraj Vadivel *et al.* 2016). Isoflavone synthase serves as the primary metabolic gateway for the production of all types of isoflavones (Cheng *et al.* 2008). Isoflavones are a plant natural product synthesized by the phenylpropanoid pathway and are exclusive to legumes (Dastmalchi and Dhaubhadel 2014). At the genome region of 37.1662 Mb to 37.1676 Mb position in IFS1, the KOR accessions exhibited low levels of nucleotide and haplotype diversity and high values of LD (Supplementary Figure 5). CYP81E11 is a gene in soybean that encodes for an enzyme known as isoflavone 2’-hydroxylase (Guttikonda *et al.* 2010). This enzyme is part of the cytochrome P450 family of monooxygenases which are involved in the biosynthesis of primary and secondary metabolites including isoflavones (Guttikonda *et al.* 2010) (Supplementary Figure 5).

We confirmed that HIDH, CYP73A11, IFS1, and CYP81E11 genes in KOR accessions have unique nucleotide and haplotype diversity patterns characterized by extended genomic regions with reduced genetic variation and high LD values. These patterns suggest selection pressure on the KOR accessions.

## Discussion

### Genomic architecture of soybean accessions

Soybeans have been domesticated and dispersed across diverse environments developing numerous locally adapted landrace soybeans. Modern breeding efforts have primarily utilized these landraces to develop improved soybeans with artificially desirable traits (Grillo 2018). Both conscious and unconscious selections have resulted in genome-wide divergence and stratification within the soybean population (Li *et al.* 2008; Zhou *et al.* 2015). Here, we investigated the population structure of 2,317 soybean whole-genome re-sequencing data. These soybean accessions included the distribution of 384 samples from SOJA, 866 from CHN, 443 from KOR, 90 from JPN, 97 from EU, 319 from NA, 62 from SA, and 56 from other countries and continents (Fig. 1a). Previous studies on soybean whole-genome sequencing have focused on understanding the genetic diversity and evolutionary history of soybean (Kajiya-Kanegae *et al.* 2021; Zhang, Jiang, *et al.* 2022). These studies have used large-scale genotyping data to examine the global distribution patterns of soybeans and to identify the genetic basis of important agronomic traits. This allows us to gain a deeper understanding of the genetic diversity and evolutionary history of soybeans and identify novel genetic variants associated with important agronomic traits.

PCA of Glycinemax revealed distinct clusters for CHN, KOR, and NA accessions despite a broad overlap among regions (Fig. 2). This cluster separation highlights the unique genetic makeup of Korean accessions, even compared to their geographically close neighbors in China. The distinct clustering of Korean accessions suggests potential avenues for further research into their unique genetic characteristics.

To further explore genetic diversity within populations, we analyzed Watterson’s theta ($\theta_{W}$) as a metric for gauging genetic diversity. $\theta_{W}$ results revealed SOJA with the highest $\theta_{W}$ value followed by moderate levels in CHN and KOR accessions. Conversely, JPN, EU, NA, and SA accessions exhibited lower $\theta_{W}$ values (Supplementary Table 4). In population genetics, an elevated $\theta_{W}$ value indicates an increased prevalence of segregating site-specific locations within the DNA sequence where genetic variations manifest. This implies that CHN and KOR accessions harbor more variable sites, suggesting heightened genetic diversity within these populations. The observed high genetic diversity in CHN accessions aligned with the notion that it supports the origins of soybean domestication in China (Hyten *et al.* 2006). Also, the relatively high genetic diversity in KOR accessions reflected characteristics associated with a lower level of artificial selection pressure and the enduring adaptation of landraces to the local environment over an long period.

Our analysis of the genetic relationship tree revealed that the KOR accessions formed a well-defined cluster within the overall *Glycine max* accessions (Fig. 1b). Furthermore, the admixture analysis demonstrated that the KOR accessions exhibited a unique genomic composition distribution compared to other *Glycine max* accessions, particularly after K=2 (Fig. 1c). Our study also discovered that the KOR accessions possessed a high level of $\theta_{W}$ and a relatively low inbreeding coefficient and LD.

Taken together, the population structure analysis and PCA, various genetic diversity measures all point towards a distinct cluster for KOR accessions, despite their geographic proximity to CHN. This finding suggests inherent genetic differentiation between the two regions. Additionally, KOR accessions exhibited the high level of $\theta_{W}$ among all analyzed *Glycine max* accessions, indicative of substantial genetic diversity. Furthermore, the genetic relationship tree, admixture analysis, and low inbreeding coefficient collectively point towards unique genetic characteristics in KOR accessions. This observed distinctiveness likely arises from long-term adaptation to the specific environmental pressures of the Korean peninsula coupled with minimal artificial selection pressure during domestication.

### Adaptive characteristics of soybean accessions

Soybean accessions have evolved their own adaptive traits that have improved their fitness, desirable agronomic traits, morphological characteristics, and ability to tolerate biotic and abiotic stresses due to environmental pressures and artificial selection. The adaptive traits found in available germplasm sets serve as valuable genetic material and resources (Hyten *et al.* 2006). From this perspective, we performed comprehensive analyses focused on selection signal analysis and KEGG pathway enrichment analysis. We identified the selection signatures of the KOR accessions which is involved in isoflavone synthesis.

According to the USDA Food Database for the isoflavone content, previous study compared the isoflavone levels in soybean populations from China (118.28 mg/100 g), Japan (130.65 mg/100 g), the European Union (103.56 mg/100 g), the United States (159.98 mg/100 g) and Brazil (99.82 mg/100 g), and found that the KOR accessions had the highest isoflavone content at 178.81 mg/100 g (Bhagwat *et al.* 2008). Our results confirmed that the KOR accessions had the highest isoflavone content among those from China, Japan, Europe, Brazil, and the United States. This suggests that the KOR accessions may be a valuable genetic resource for improving isoflavone production in soybean breeding programs or for studying the genetic mechanisms underlying isoflavone synthesis.

The unique genetic and physiological characteristics observed in the KOR accessions suggest that it has undergone local adaptation to the environmental conditions of the Korean Peninsula. The climate of the Korean Peninsula is known for its significant temperature differences, distinct rainy and dry seasons, and extreme temperature fluctuations. In the summer, the maximum temperature reaches up to 40∘C, accompanied by about 70% of the average annual precipitation. In contrast, the winter brings cold and dry conditions with a minimum temperature of about $-32^{\circ }$C (KMA 2023). These environmental factors exert strong pressure on the local animal (Kim *et al.* 2019; Cho *et al.* 2022) and plant populations potentially leading to positive selection and shaping the genetic makeup of the KOR accessions over time. Isoflavones which are a subclass of flavonoid compounds found in soybeans possess various biological activities that may contribute to plant resistance to adverse environments. During periods of extreme weather such as drought or high temperatures, plants often experience water stress and oxidative damage. Isoflavones have been found to accumulate in the root elongation zone under drought-stress conditions and act as osmolytes to help maintain cell turgor and protect the plant from the effects of water stress (Yamaguchi *et al.* 2010). This antioxidant activity may mitigate the oxidative damage caused by extreme weather conditions and help plants withstand environmental stressors (Trush and Pal’ove-Balang 2023).

In addition, isoflavones have attracted attention for potential health benefits. Consumption of soy isoflavones has shown potential in reducing cholesterol levels and exhibiting anticancer properties in several previous studies (Sarkar and Li 2004; Taku *et al.* 2007). Additionally, soy isoflavones have demonstrated immune enhancement, anti-aging effects, and other pharmacological benefits (Kim 2021; Al-Shaikh 2022). The selection signature observed in the KOR accessions is specifically associated with isoflavone synthesis suggesting that these compounds play an important role as phytoalexins, which are natural defense compounds that provide health benefits to both humans and plants (Treutter 2005; Sohn *et al.* 2021).

In keeping with our objective of identifying valuable soybean accessions for future breeding studies and programs, this study provides statistical evidence of the adaptive traits of the KOR accessions and their related candidate genes. However, our results have several limitations. First, one limitation of our study is the variability in sample size among soybean accessions, which may impact the statistical analysis. However, with the increasing availability of open whole-genome sequencing data, this issue may be mitigated in future studies. Second, our results indicate potential variability within soybean accessions. This observation aligns with our hypothesis that population stratification and genomic differences have arisen due to selection, adaptation and bottleneck events. Third, while our results identified a signature of selection in KOR accessions, these findings have not been validated through biological experimentation. We employed rigorous statistical methods to address this limitation and applied conservative thresholds in our genome analysis.

Overall, our study provides valuable information for soybean breeders to improve soybean productivity in changing climate patterns and new locations by identifying germplasms that can serve as donor parents. This knowledge on adaptation at the SNP level can also aid in the genomic prediction of yield under specific environmental conditions (Lasky *et al.* 2015). However, further validation is needed through common garden experiments and gene editing confirmation. This research approach could enhance our comprehension of soybean adaptation and facilitate the extraction of beneficial alleles from germplasm collections. Additionally, the proposal that KOR accessions have selection signatures on isoflavone to protect from extreme environmental stress is supported by genetic, physiological, and ecological evidence. Further experimental research is necessary to fully comprehend the mechanisms and implications of this adaptation and explore the potential applications of isoflavone in crop improvement and environmental management. Nonetheless, the candidate genes and their associated genomic regions that have been identified can provide valuable insights for future research aimed at utilizing the specific adaptive traits of each soybean accession.

## Supplementary Material

jkae118_Supplementary_Data

## Data Availability

Publicly available data from the SRA, utilized as the primary source in this study, are summarized in Supplementary File 1. The code to reproduce the pangenome from this work can be found at GitHub (https://github.com/skyclub3/SoyPopGeneticsPipeline). Supplemental material available at G3 online.
