# Supplementary material for: Whole-genome sequencing analysis of soybean diversity across different countries and selection signature of Korean soybean accession
Source: G3 (Bethesda). 2024 Jun 4;14(8):jkae118. doi: 10.1093/g3journal/jkae118 (PMC11304964; doi:10.1093/g3journal/jkae118)
Supplement: jkae118_Supplementary_Data [file jkae118_supplementary_data.zip › Supplemental_Material_G3-2023-404786.pdf]

Supplementary Material

1 FIGURES

Figure S1. The scatter plot provides a comprehensive visualization of the characteristics of soybean variants utilized in the Variant Call Format (VCF) filtration criteria. The x-axis of the plot signifies the proportion of Single Nucleotide Polymorphisms (SNPs), whereas the y-axis encapsulates variant annotations. These annotations include QualByDepth (QD), FisherStrand (FS), MappingQualityRankSumTest (MQRankSum), ReadPosRankSumTest (ReadPosRankSum), Mapping Quality (MQ), and Mean Depth (DP). The red dotted line demarcates our specific VCF filtration criteria.

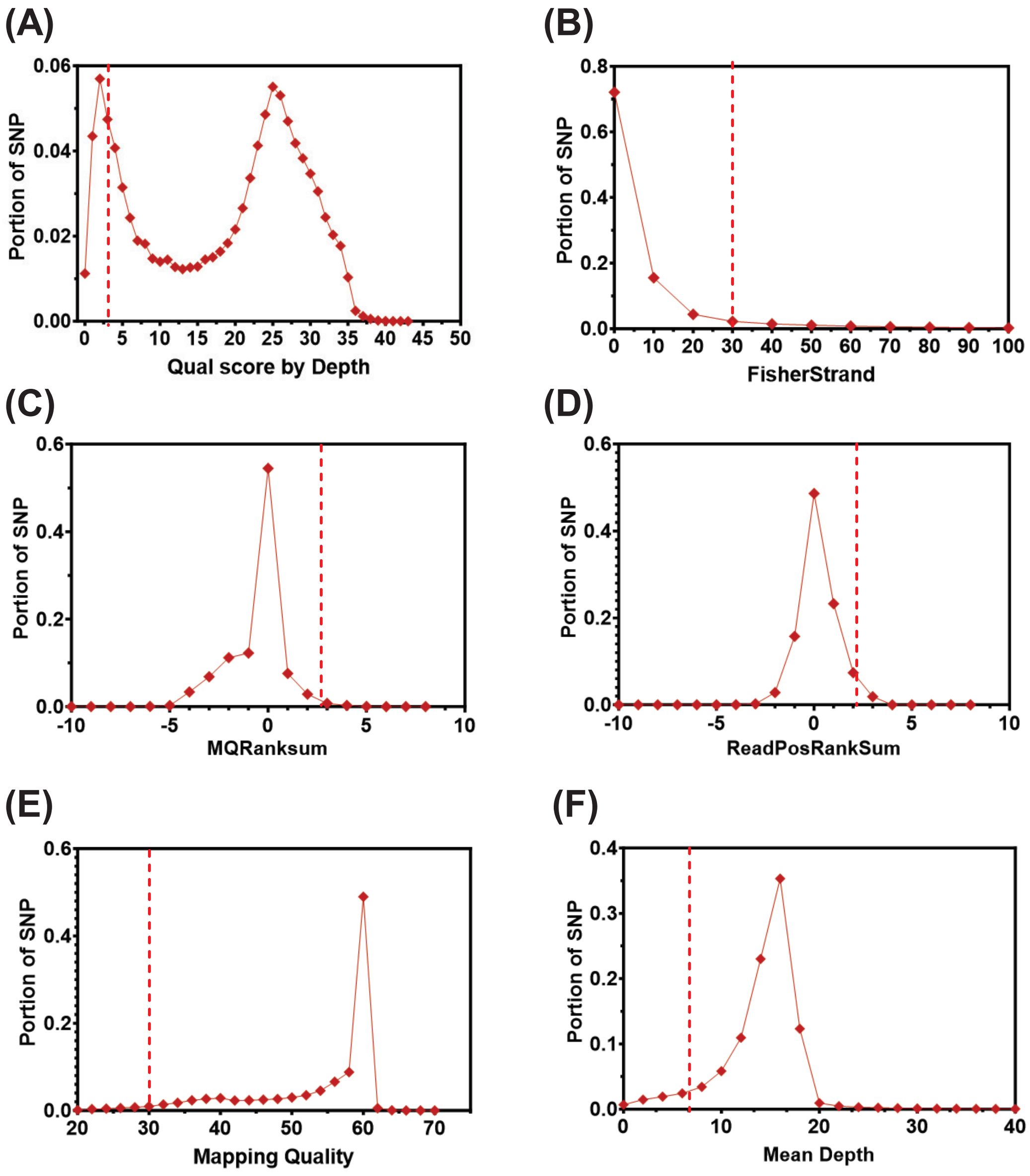

Figure S2. The scatter plots offers a detailed visualization of the characteristics of soybean variants, as determined by the VCF filtration criteria. The red dotted line signifies the threshold set by our VCF filtration process, providing a clear demarcation for variant selection.

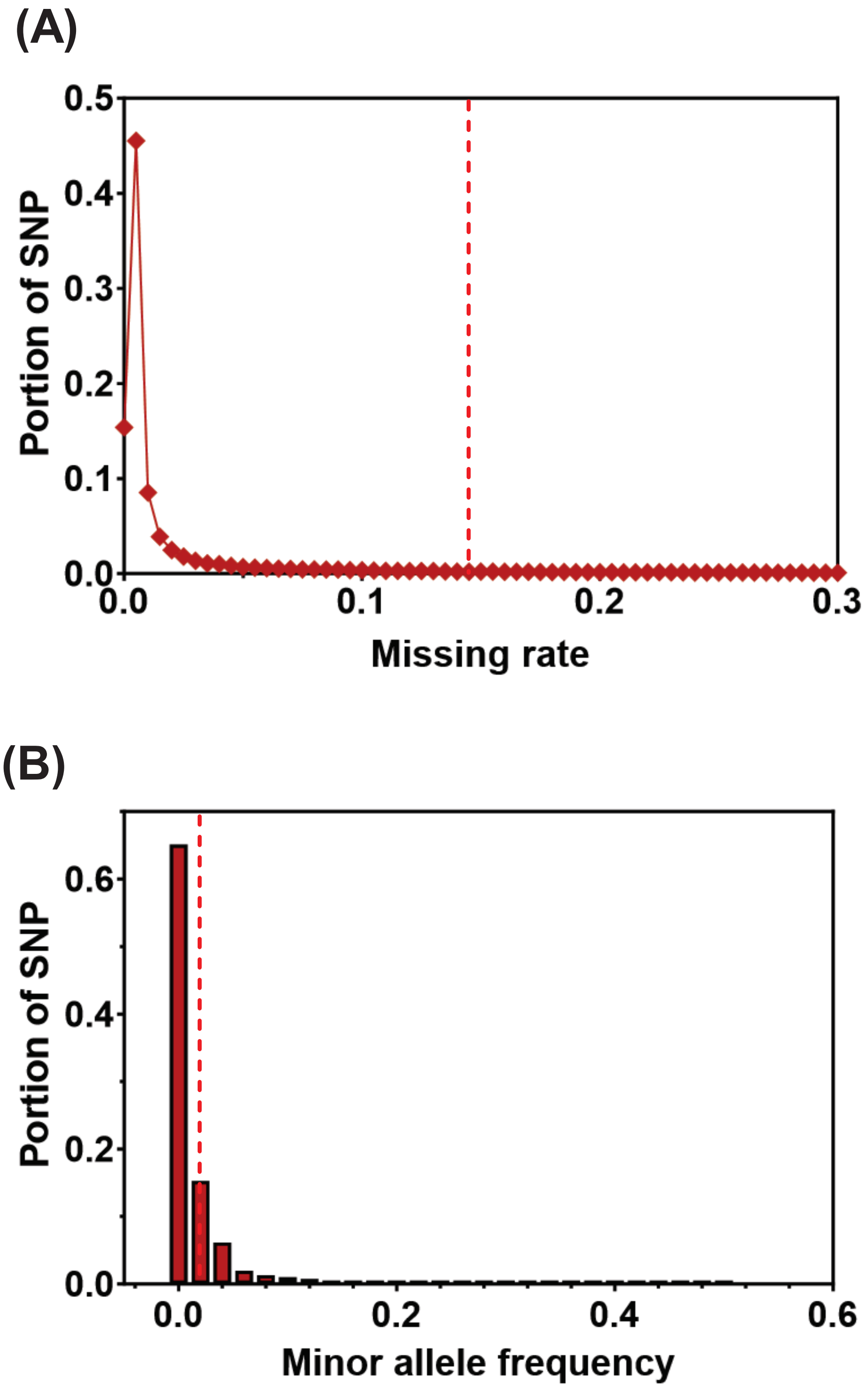

Figure S3. A histogram displaying the number of bi-allelic SNPs identified in the soybean samples.

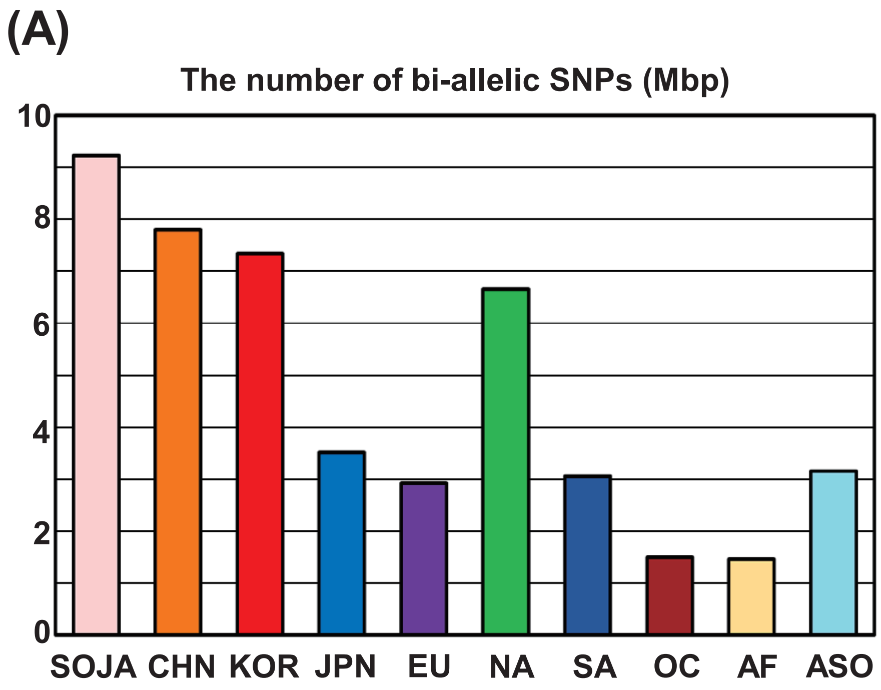

Figure S4. Selection signature for isoflavone synthesis in KOR soybean accession. (A-B) The degree of nucleotide and haplotype diversity, as well as linkage disequilibrium (top), and the extent of haplotype sharing among all accessions (bottom) are shown for Glyma_01G239600 and Glyma_02G236500. Nucleotide and haplotype diversity are displayed for all 7 accession lines (C-D) The figure displays gene structures and haplotype frequencies for regions containing one missense variant in two soybean genes, Glyma_01G239600 and Glyma_02G236500. The gene structures are shown on the top, and the regions containing the missense variant and its surrounding 14 variants are displayed below them. The reference and alternative alleles are indicated in green and yellow, respectively, and the missense variants are highlighted in light yellow. The missense variants of Glyma_01G239600 and Glyma_02G236500 are located at 56,367,506 bp on chromosome 1 and at 42,416,516 bp on chromosome 2, respectively. The haplotype structures for those regions are depicted below the gene structures, and the haplotype frequencies are presented to the right. Due to illustration constraints, only the top two haplotype frequencies are shown for each accession.

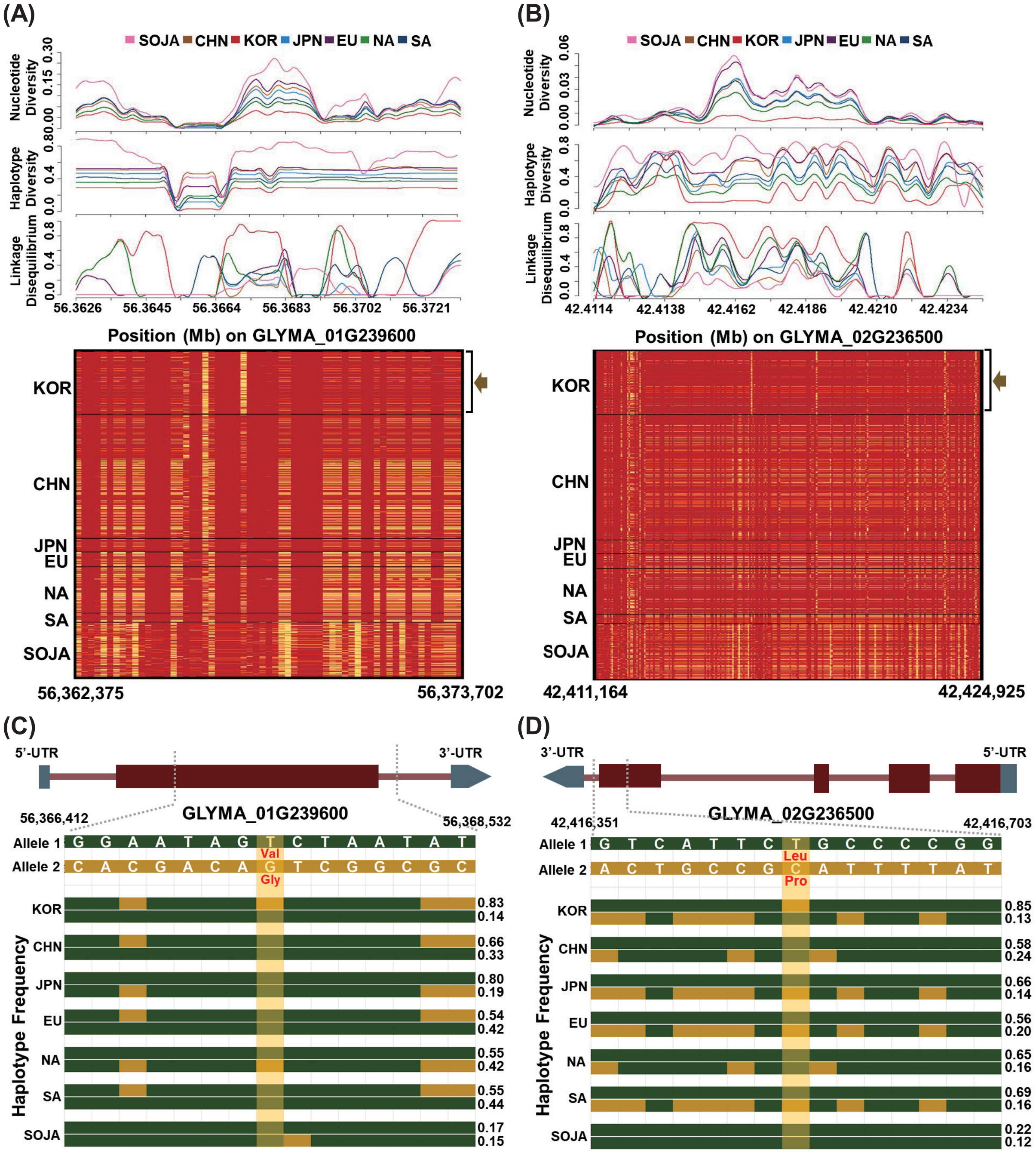

Figure S5. Selection signature for isoflavone synthesis in KOR soybean accession. (A-B) The degree of nucleotide and haplotype diversity, as well as linkage disequilibrium (top), and the extent of haplotype sharing among all accessions (bottom) are shown for Glyma_07G202300 and Glyma_09G049100. Nucleotide and haplotype diversity are displayed for all 7 accession lines (C-D) The figure displays gene structures and haplotype frequencies for regions containing one missense variant in two soybean genes, Glyma_07G202300 and Glyma_09G049100. The gene structures are shown on the top, and the regions containing the missense variant and its surrounding 14 variants are displayed below them. The reference and alternative alleles are indicated in green and yellow, respectively, and the missense variants are highlighted in light yellow. The missense variants of Glyma_07G202300 and Glyma_09G049100 are located at 37,166,934 bp on chromosome 7 and at 42,65,280 bp on chromosome 9, respectively. The haplotype structures for those regions are depicted below the gene structures, and the haplotype frequencies are presented to the right. Due to illustration constraints, only the top two haplotype frequencies are shown for each accession.

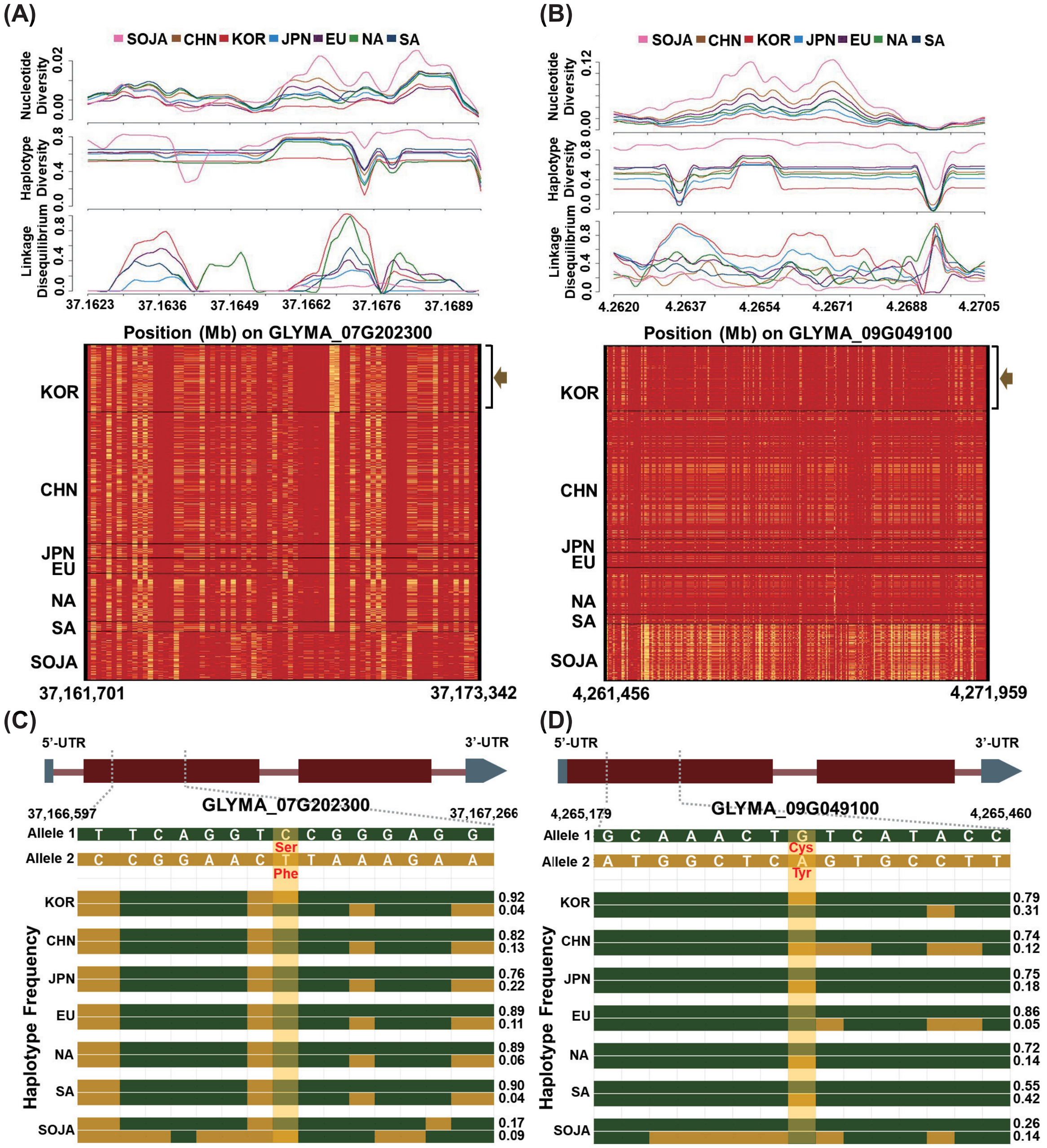

2 TABLE

Table S1. Comprehensive Summary of *Glycine max* Samples Collected

Country	Continent	iso_alpha	Number of Sample
Algeria	Africa	DZA	5
Argentina	SouthAmerica	ARG	2
Australia	Oceania	AUS	3
Austria	EU	AUT	2
Belgium	EU	BEL	1
Brazil	SouthAmerica	BRA	59
Bulgaria	EU	BGR	1
Canada	NorthAmerica	CAN	36
China	Asia	CHN	866
France	EU	FRA	5
Georgia	EU	GEO	1
Germany	EU	DEU	6
Hungary	EU	HUN	6
Indo	Asia	IND	13
Indonesia	Asia	IDN	6
Japan	Asia	JPN	90
Korea	Asia	KOR	443
Moldova	EU	MDA	9
Morocco	Africa	MAR	1
Myanmar	Asia	MMR	3
Nepal	Asia	NPL	2
Netherlands	EU	NLD	2
Pakistan	Asia	PAK	1
Peru	SouthAmerica	PER	1
Philippines	Asia	PHI	3
Poland	EU	POL	1
Romania	EU	ROM	7
Russia	EU	RUS	42
Serbia	EU	SER	6
South Africa	Africa	ZAF	1
Sweden	EU	SWE	5
Taiwan	Asia	TWN	6
Tanzania	Africa	TZA	1
Thailand	Asia	THA	1
Ukraine	EU	UKR	3
USA	NorthAmerica	USA	283
Uzbekistan	Asia	UZB	1
Viet Nam	Asia	VNM	9

Table S2. Number of variants detected across chromosomes in a dataset of 2,317 soybean accession samples. The table presents the distribution of genetic variants along the different chromosomes, providing insights into the genetic diversity within the soybean population.

Chromosome	Length(bp)	Numbers of total variants	Numbers of filtered variants	Numbers of SNPs	Numbers of INDEL variants	Average distance between SNPs
1	57,977,878	292,120,808	274,719,309	141,484,290	133,235,019	2.06
2	50,461,126	224,850,843	211,753,573	108,143,769	103,609,804	2.08
3	47,399,459	236,217,985	222,888,604	115,663,422	107,225,182	2.04
4	51,203,389	245,784,852	231,553,270	117,254,872	114,298,398	2.10
5	42,406,958	207,639,611	195,459,292	96,922,619	98,536,673	2.14
6	51,363,926	253,553,555	239,393,493	120,248,969	119,144,524	2.11
7	45,063,974	225,164,923	212,315,563	110,337,639	101,977,924	2.04
8	47,232,357	211,176,528	199,130,532	106,910,963	92,219,569	1.98
9	50,618,741	233,282,733	219,871,307	111,623,151	108,248,156	2.09
10	51,843,406	246,118,418	231,899,656	117,540,884	114,358,772	2.09
11	39,734,517	174,392,848	164,166,607	85,576,793	78,589,814	2.04
12	41,547,854	199,134,876	187,473,673	95,906,205	91,567,468	2.08
13	46,186,682	210,027,506	198,321,139	105,332,254	92,988,885	1.99
14	50,003,496	251,155,973	236,605,927	118,365,871	118,240,056	2.12
15	53,835,918	272,424,149	257,120,928	131,730,473	125,390,455	2.07
16	38,143,498	209,127,616	197,716,433	101,076,136	96,640,297	2.07
17	41,740,656	202,421,655	182,343,314	95,731,997	86,611,317	2.11
18	58,458,914	315,769,139	298,203,748	145,773,648	152,430,100	2.17
19	51,384,346	240,373,886	226,424,553	114,681,142	111,743,411	2.10
20	47,898,032	240,381,744	226,667,729	113,893,675	112,774,054	2.11

Table S3. Summary statistics of SNP data, including the total number of SNPs, average number of SNPs per sample, standard deviation of SNPs per sample, and the total number of bi-allelic SNPs. The table provides an overview of the SNP characteristics, offering insights into the genetic variation present in the dataset.

Group	Total number of SNP	Average number of SNP each sample	Standard deviation of SNP each sample	Total number of bi-SNP
SOJA	331,580,994	670,541	116,409	9,223,755
CHN	270,075,920	357,424	73,975	7,798,075
KOR	221,600,379	337,834	62,735	7,342,058
JPN	85,278,279	323,452	58,599	3,512,283
EU	89,854,917	315,094	58,126	2,930,790
NA	251,908,999	283,418	86,328	6,649,742
SA	227,234,171	300,106	50,579	3,059,564
OC	11,031,812	356,057	19,282	1,490,560
AF	15,646,426	279,848	90,107	1,459,043
ASOTHER	44,432,796	384,540	100,972	3,154,916

Table S4. Comparative Analysis of Nucleotide Diversity (θ_W), Inbreeding coefficient (F), and Linkage disequilibrium (LD) Across Different Soybean Accessions

**Group**	θ_W	**F**	r^2 **(500Kb)**
SOJA (n=384)	1.94×10^{-3}	8.59×10^{-1}	2.07×10^{-1}
CHN (n=866)	1.75×10^{-3}	8.76×10^{-1}	3.76×10^{-1}
KOR (n=443)	1.50×10^{-3}	8.84×10^{-1}	4.01×10^{-1}
JPN (n=90)	1.20×10^{-3}	9.00×10^{-1}	4.18×10^{-1}
EU (n=97)	1.18×10^{-3}	8.96×10^{-1}	4.24×10^{-1}
NA (n=319)	1.01×10^{-3}	8.77×10^{-1}	3.89×10^{-1}
SA (n=62)	0.92×10^{-3}	8.87×10^{-1}	4.90×10^{-1}

Table S5. Comparison of pairwise Fst scores among soybean accessions to assess population structure.

Group	SOJA	CHN	KOR	JPN	EU	NA	SA
SOJA	0.00	4.81×10^{-2}	5.70×10^{-2}	8.06×10^{-2}	7.35×10^{-2}	6.15×10^{-2}	8.15×10^{-2}
CHN	4.81×10^{-2}	0.00	2.42×10^{-2}	2.14×10^{-2}	3.18×10^{-2}	3.27×10^{-2}	3.35×10^{-2}
KOR	5.70×10^{-2}	2.42×10^{-2}	0.00	2.55×10^{-2}	2.94×10^{-2}	3.02×10^{-2}	3.29×10^{-2}
JPN	8.06×10^{-2}	2.14×10^{-2}	2.55×10^{-2}	0.00	1.79×10^{-2}	2.46×10^{-2}	1.87×10^{-2}
EU	7.35×10^{-2}	3.18×10^{-2}	2.94×10^{-2}	1.79×10^{-2}	0.00	2.85×10^{-2}	2.47×10^{-2}
NA	6.15×10^{-2}	3.27×10^{-2}	3.02×10^{-2}	2.46×10^{-2}	2.85×10^{-2}	0.00	2.17×10^{-2}
SA	8.15×10^{-2}	3.35×10^{-2}	3.28×10^{-2}	1.87×10^{-2}	2.47×10^{-2}	2.17×10^{-2}	0.00
